# Increased expression of secreted frizzled related protein 1 (SFRP1) predicts ampullary adenocarcinoma recurrence

**DOI:** 10.1038/s41598-020-69899-8

**Published:** 2020-08-06

**Authors:** Li-Chin Cheng, Ying- Jui Chao, Michael J. Overman, Chih -Yang Wang, Nam Nhut Phan, Yi-Ling Chen, Tzu-Wen Wang, Hui-Ping Hsu, Yan-Shen Shan, Ming- Derg Lai

**Affiliations:** 1 Division of Colorectal Surgery, Department of Surgery, Chi-Mei Medical Center, Tainan, Taiwan; 2 Department of Surgery, National Cheng Kung University Hospital, College of Medicine, National Cheng Kung University, No. 138, Sheng-Li Rd., Tainan, 70403 Taiwan; 3 Institute of Clinical Medicine, College of Medicine, National Cheng Kung University, Tainan, Taiwan; 4 Department of Gastrointestinal Medical Oncology, The University of Texas MD Anderson Cancer Center, Houston, TX USA; 5 Department of Biochemistry and Molecular Biology, College of Medicine, National Cheng Kung University, Tainan, Taiwan; 6 Institute of Basic Medical Sciences, College of Medicine, National Cheng Kung University, Tainan, Taiwan; 7 Ph.D. Program for Cancer Molecular Biology and Drug Discovery, College of Medical Science and Technology, Taipei Medical University, Taipei, Taiwan; 8 Graduate Institute of Cancer Biology and Drug Discovery, College of Medical Science and Technology, Taipei Medical University, Taipei, Taiwan; 9 NTT Institute of Hi-Technology, Nguyen Tat Thanh University, Ho Chi Minh City, Vietnam; 10 Senior Citizen Service Management, Chia-Nan University of Pharmacy and Science, Tainan, Taiwan; 11 Department of Biostatistics, Vanderbilt University Medical Center, Nashville, TN USA; 12 Center for Infectious Diseases and Signaling Research, College of Medicine, National Cheng Kung University, Tainan, Taiwan

**Keywords:** Gastrointestinal cancer, Oncogenes

## Abstract

Ampullary adenocarcinoma is a rare gastrointestinal cancer in which WNT signalling dysregulation has been previously reported. Secreted frizzled related protein 1 (SFRP1) is one of the extracellular ligands of WNT signalling. We performed bioinformatics analyses of SFRP1 expression in human cancer. Microarray analysis of SFRP1 in periampullary adenocarcinoma was obtained from the Gene Expression Omnibus GSE39409 dataset. SFRP1 expression in ampullary adenocarcinoma was detected by immunohistochemistry staining and correlated with patients’ clinical outcomes. Our results showed that SFRP1 expression had different clinical applications in all types of human cancer. No detected alteration of *SFPR1* gene and SFRP1 expression in ampullary adenocarcinoma was lower than that in other periampullary adenocarcinomas. However, high expression levels of SFRP1 protein were correlated with cancer recurrence, peritoneal carcinomatosis and poor patient prognosis. Gene set enrichment analysis showed downregulation of multiple WNT-related genes in primary culture cells from ampullary adenocarcinoma, but SFRP1 expression was increased. We found an interaction between WNT, bone morphogenetic protein and hedgehog signalling with SFRP1. Furthermore, a high expression of SFRP1 predicted poor prognosis for ampullary adenocarcinoma patients. Because it is a multifunctional protein, SFRP1 targeting serves as a potential therapy for ampullary adenocarcinoma patients.

## Introduction

The incidence of periampullary adenocarcinoma is low. Ampullary adenocarcinoma, a subset of periampullary adenocarcinoma, has a high rate of resectability^1^. The ampulla of Vater is located in second portion of the duodenum at the orifice of the common channel, the conjunction of pancreatic duct and common bile duct. The mucosa of the ampulla of Vater is continuously exposed to an alkaline environment, contacted with enzymes from pancreatic juice, toxins from bile juice, and microbiota in duodenal juice. This complex environment could account for the development of ampullary cancer^2^. Adenocarcinoma is the most common histological type of ampullary cancer^3^. Ampullary adenocarcinoma can be subcategorised into pancreaticobiliary and intestinal subtypes^4^. Patients with pancreaticobiliary subtypes have a worse prognosis than those with intestinal subtypes. Furthermore, the carcinogenesis of ampullary cancer involves aberrant activation of WNT signalling^5^. The canonical WNT-β-catenin pathway begins with the extracellular ligands, the WNT proteins. The binding of WNT proteins to membrane receptors activates the phosphorylation cascade of downstream molecules, including frizzled receptors, which are transmembranous G protein-coupled receptors, and β-catenin, the intracellular representative protein of the WNT pathway.

The canonical pathway of WNT activation in cancer cells is the accumulation of intracellular and nuclear β-catenin due to a loss of chaperon proteins^6^. The movement of β-catenin into the nucleus results in the transcription of a series of genes involved in cell cycle progression^7^. β-catenin accumulation has been reported in ampullary adenocarcinoma, particularly in the intestinal subtypes^8^. Genetic mutation of WNT signalling has been observed in ampullary and periampullary cancer, including *APC* (adenomatosis polyposis coli), *AXIN1*, *SOX9*, and *FBXW7*^5,9,10^. *CTNNB1* mutation (catenin beta 1, β-catenin protein) induces the nuclear accumulation of β-catenin in ampullary cancer^11^. In contrast, the loss of β-catenin has been correlated with poor prognosis for ampullary cancer patients^12,13^. Other mutations have been reported in gastrointestinal and colorectal cancer, such as mutation of *APC*, *CTNNB1*, *RNF43* (ring finger protein 43), *WNT1* (WNT family member 1), and *CDH1* (cadherin 1, E-cadherin)^14–16^. Thus, the aberrant or non-canonical regulation of WNT signalling possibly contributes to ampullary cancer development.

Secreted frizzled related protein 1 (SFRP1) belongs to the SFRP family of proteins with a cysteine-rich domain. The cysteine-rich domain mimics the WNT-binding site of frizzled receptor^17^. SFRP family proteins act as modulators of WNT signalling. SFRP1 inhibits WNT-dependent transcription and decreases the intracellular level of β-catenin^18^. The majority of studies have reported that SFRP1 loss is correlated with poor prognosis in breast cancer, lung cancer, cholangiocarcinoma, and hepatocellular carcinoma^19–22^. The methylation of SFRP1 promoter induces gene silencing and has been detected in 29 cancer types^23,24^. However, some studies contradict these findings. In metastatic renal cell carcinoma, *SFRP1* hypomethylation increases SFRP1 expression and activates histone modifications^25^. Overexpression of SFRP1 protein is related to cell line invasiveness^25^. Furthermore, it is correlated with lymph node metastasis, cell proliferation, epithelial–mesenchymal transition (EMT), invasion, and poor survival of gastric cancer patients^26^. The complex function of SFRP1 in human cancer requires further investigation. Ampullary cancer originates from complex environment and the mechanism of carcinogenesis is obscure. In the present study, we utilised bioinformatics analysis to investigate SFRP1 function in multiple types of cancers. Then, we studied protein and mRNA expression in clinical samples from patients with ampullary adenocarcinoma to verify the function of SFRP1 in ampullary adenocarcinoma.

## Results

### SFRP1 expression in different cancer types

**Figure 1:**
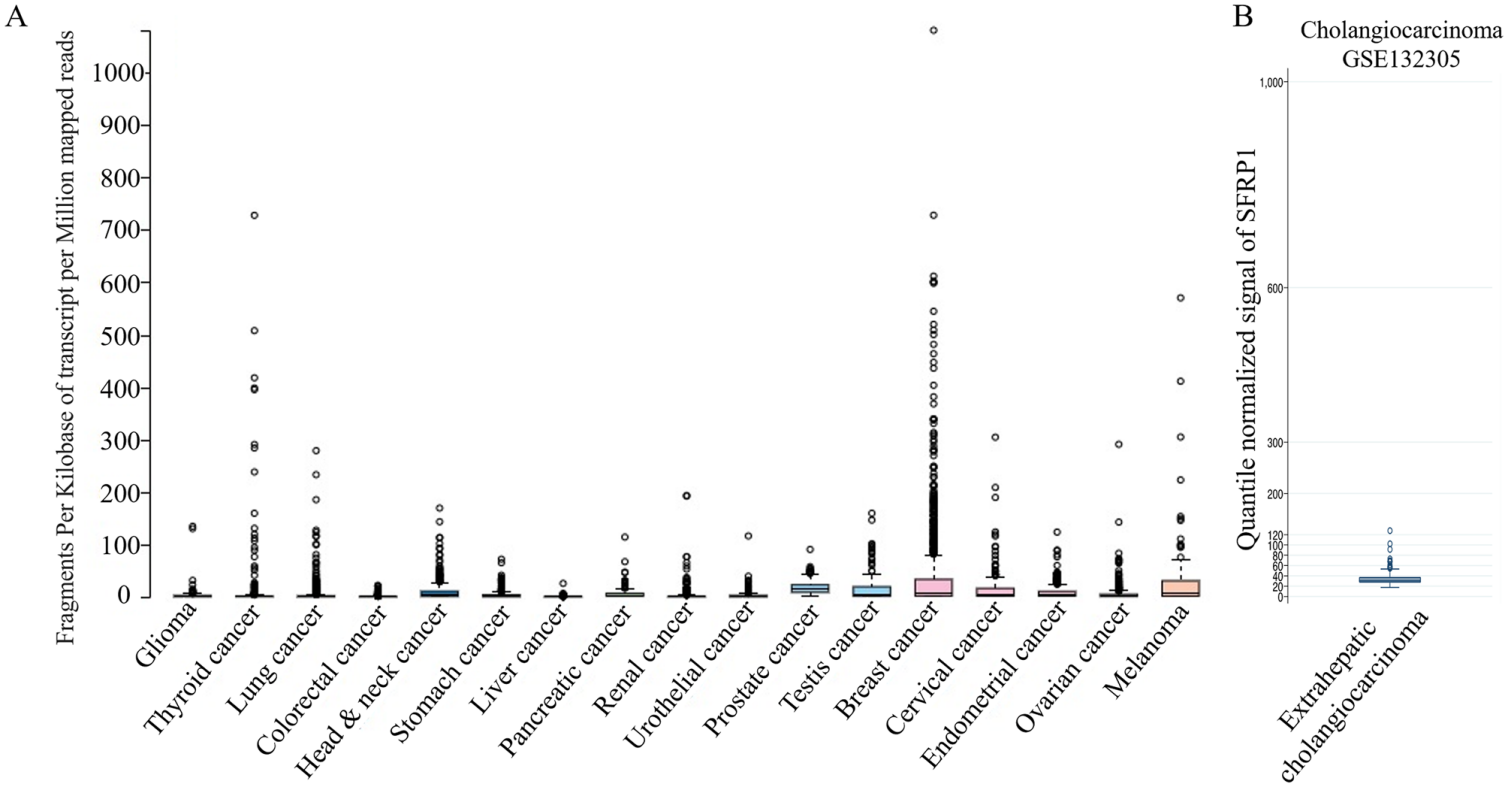
Expression of SFRP1 mRNA in different datasets. (**A**) SFRP1 expression in pan-cancer atlas of The Cancer Genome Atlas (TCGA), extracted from The Human Protein Atlas programme. (**B**) Expression of SFRP1 in extrahepatic cholangiocarcinoma from the GSE132305 dataset. Expression level of SFRP1 is different in various types of cancer. SFRP1, secreted frizzled related protein 1.

**Figure 2:**
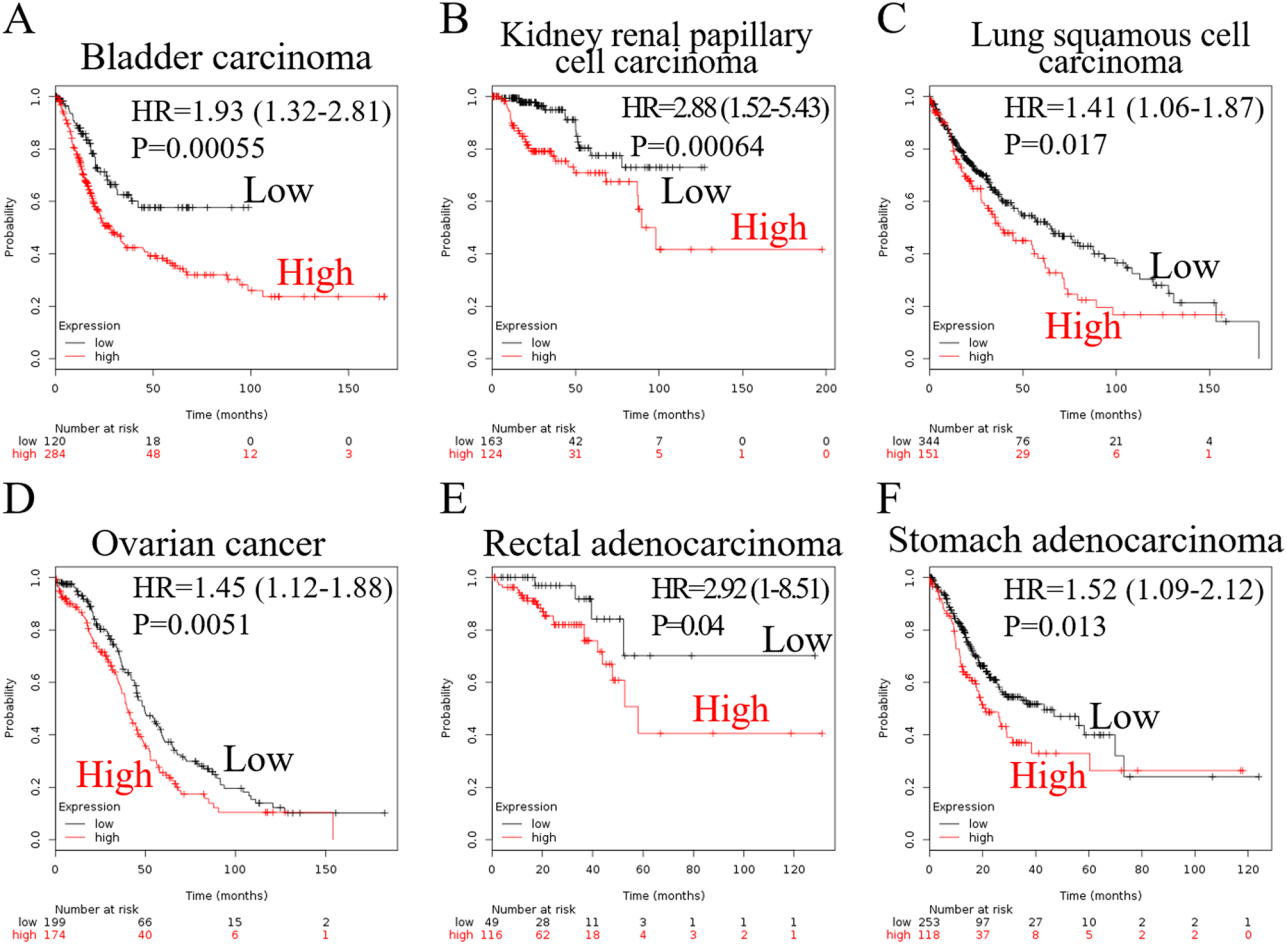
Assessment of the effect of SFRP1 mRNA expression by Kaplan–Meier Plotter. High expression of SFRP1 was correlated with poor prognosis in patients with (**A**) bladder carcinoma, (**B**) kidney renal papillary cell carcinoma, (**C**) lung squamous cell carcinoma, (**D**) ovarian cancer, (**E**) rectal adenocarcinoma, and (**F**) stomach adenocarcinoma. SFRP1, secreted frizzled related protein 1; HR, hazard ratio.

**Figure 3:**
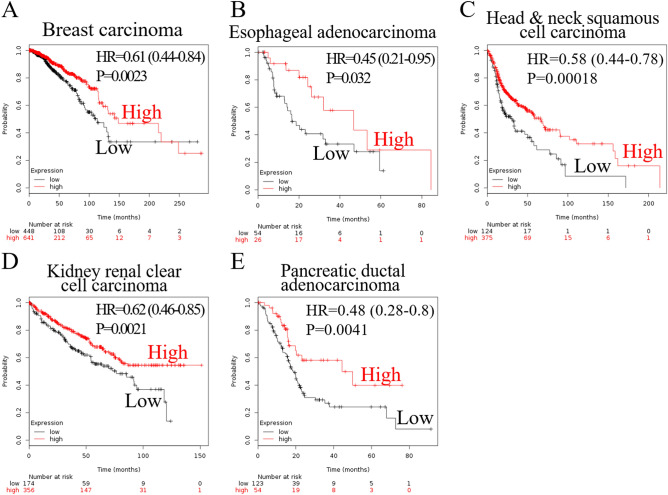
Assessment of the effect of SFRP1 mRNA expression by Kaplan–Meier Plotter. Low expression of SFRP1 was correlated with poor prognosis in patients with (**A**) breast carcinoma, (**B**) esophageal adenocarcinoma, (**C**) head and neck squamous cell carcinoma, (**D**) kidney renal clear cell carcinoma, and (**E**) pancreatic ductal adenocarcinoma. SFRP1, secreted frizzled related protein 1; HR, hazard ratio.

The expression level of *SFRP1* mRNA was diverse (Fig. 1A), suggesting that function of SFRP1 may be distinct in different types of cancer. A microarray study of 182 extrahepatic cholangiocarcinomas detected *SFRP1* expression (Fig. 1B). Because ampullary adenocarcinoma is rare, there was no data available in The Human Protein Atlas. To verify the difference of SFRP-associated phenotypes, we analysed a pan-cancer panel of Kaplan–Meier Plotter based on 21 cancer types. The probability of survival was analysed according to expression level of SFRP1 mRNA. The correlation between high SFRP1 expression with patients’ survival was diverse and was correlated with poor prognoses in bladder carcinoma, kidney renal papillary cell carcinoma, lung squamous cell carcinoma, ovarian cancer, and rectal and stomach adenocarcinoma (Fig. 2). However, a high expression level of SFRP1 was associated with better prognosis in patients with breast carcinoma, esophageal adenocarcinoma, head and neck squamous cell carcinoma, kidney renal clear cell carcinoma, and pancreatic ductal adenocarcinoma (Fig. 3). In 10 other cancer types, the correlation between SFRP1 expression and patient survival was weak, with a wide 95% confidence interval (CI) (Supplementary Fig. S1).

There are several microarray panels available with variable detection sensitivity. The PrognoScan database is a collection of multiple array types in an abundant number of cancers. The expression of SFRP1 was divided into high or low level of mRNA. The Cox proportional hazard ratio (HR) was calculated to obtain a *P*-value. We selected datasets where the *P*-value was < 0.05. The expression of SFRP1 and cancer prognosis are presented in Supplementary Table S1 and Supplementary Table S2. The results from datasets of lung adenocarcinoma were controversial. Low SFRP1 was correlated with poor prognosis of patients in one of the datasets (GSE31210), and high SFRP1 was associated with poor prognosis in a different dataset (jacob-00182-CANDF). Moreover, the result from PrognoScan in patients with head and neck squamous cell carcinoma (GSE2837) was completely different from the overall survival in Kaplan–Meier Plotter (Fig. 3). The results of the former dataset showed that high SFRP1 expression was associated with a poor prognosis, and the latter software concluded that high SFRP1 expression was correlated with a better prognosis. All these controversial results from the bioinformatics databases required additional research for clarity.

**Figure 4:**
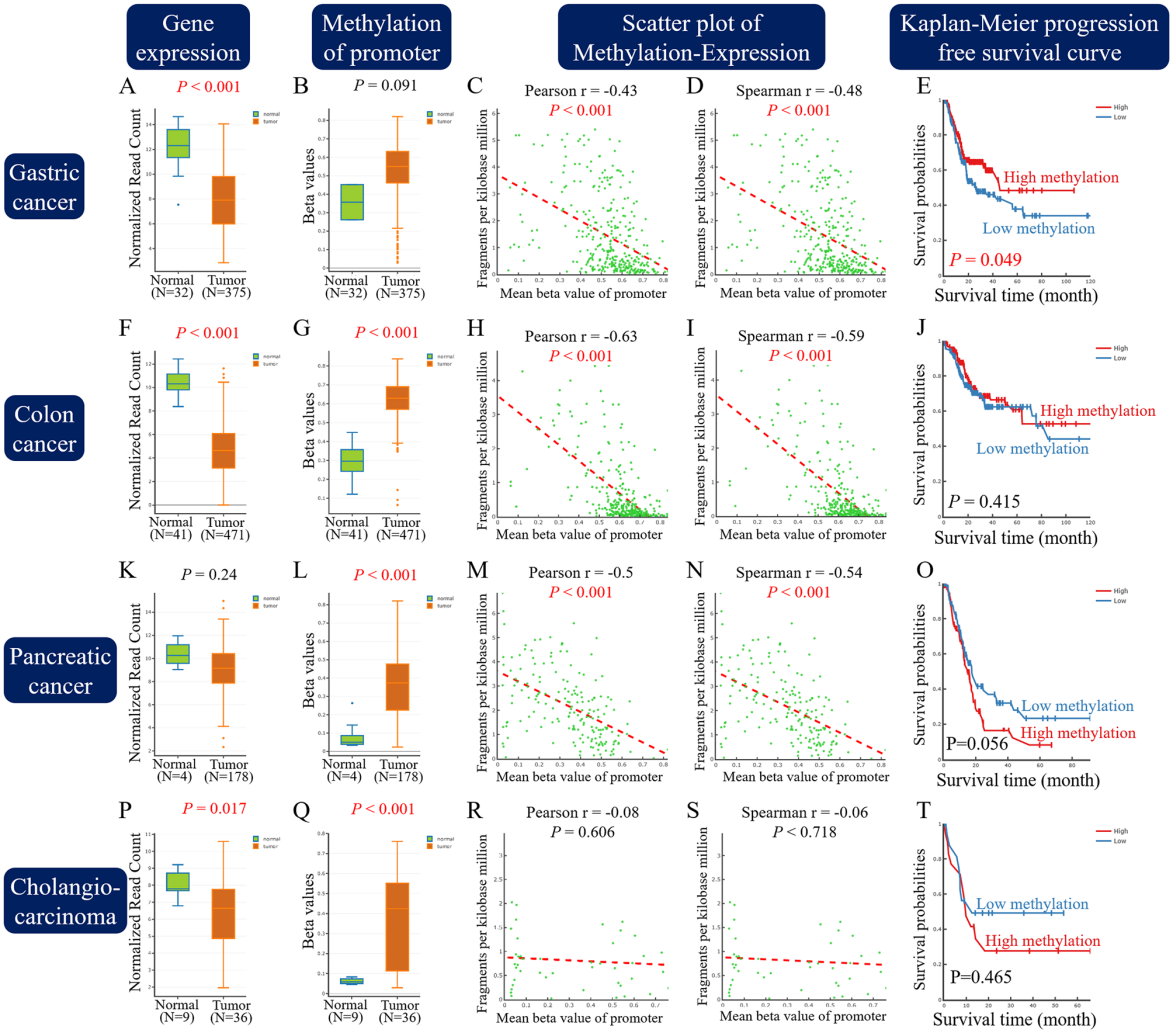
Analysis of SFRP1 in DNA methylation interactive visualisation database. Five different profiles of information was shown in four kinds of cancer. (**A**,**F**,**K**,**P**) Gene expression of SFRP1 in RNA-sequencing read count. (**B**,**G**,**L**,**Q**) Differential methylation of the promoter region of SFRP1. (**C**,**H**,**M**,**R**) Scatter plots of promoter methylation and gene expression, analysed by Pearson’s method. (**D**,**I**,**N**,**S**) Spearman’s correlation analysis of promoter methylation and gene expression. (**E**,**J**,**O**,**T**) Kaplan–Meier progression-free survival curve by the degree of methylation of SFRP1. (**A**–**E**), gastric cancer; (**F**–**J**), colon cancer. (**K**–**O)**, pancreatic cancer; (**P**–**T**), cholangiocarcinoma. SFRP1, secreted frizzled related protein 1.

Transcription regulation involves DNA methylation and histone deacetylation. Information about promoter methylation of SFRP1 was acquired from the DNA Methylation Interactive Visualisation Database (DNMIVD). We were unable to find any ampullary cancer datasets from Kaplan–Meier Plotter, PrognoScan, and DNMIVD. We selected two gastrointestinal adenocarcinoma (gastric and colon adenocarcinoma) and two periampullary adenocarcinoma (pancreatic adenocarcinoma and cholangiocarcinoma) cases to simulate ampullary adenocarcinoma (Fig. 4). SFRP1 expression and differential methylation of the promoter region were compared. An inverse ratio of gene expression (Fig. 4A,F,K,P) and promoter methylation (Fig. 4B,G,L,Q) was detected in all four types of adenocarcinoma. Pearson’s and Spearman’s correlation coefficients were calculated, and a negative correlation was confirmed in gastric, colon and pancreatic adenocarcinoma. High methylation of SFRP1 represented decreased gene expression (Fig. 4C,D,H,I,M,N). The limited number of patients or wide variation of gene expression and promoter methylation in cholangiocarcinoma resulted in failure to establish the correlation (Fig. 4R,S). The impact of DNA methylation was correlated with patients’ survival. For the patients with gastric adenocarcinoma, low methylation of SFRP1 (high expression levels of *SFRP1*) was correlated with poor patient prognosis (Fig. 4E). Low methylation of SFRP1 (high expression of *SFRP1*) showed a trend of correlation with better prognosis in patients with pancreatic adenocarcinoma and cholangiocarcinoma (Fig. 4O,T). The results of gastric and pancreatic adenocarcinoma in DNMIVD were similar with the survival correlation in Kaplan–Meier Plotter (Figs. 2F and 3E).

### Expression of WNT-related genes in different kinds of cancer

To study other WNT-related genes in four types of adenocarcinoma, we collected gene expression level from DNMIVD and mutation data from the cBioPortal platform. *APC* expression gene was decreased in gastric adenocarcinoma. The expression of *WNT1*, *APC* was decreased in colon adenocarcinoma, and that of *CTNNB1* was decreased in cholangiocarcinoma. The level of *RNF43* was increased in gastric, colon and pancreatic adenocarcinoma. The expression of *CDH1* gene was controversial (Supplementary Fig. S2). The differences in gene expression between tumor and normal tissue were relatively small, but that of SFRP1 gene expression was larger (Fig. 4).

The mutation status of these cancers and ampullary adenocarcinoma are shown in Supplementary Fig. S3 and Supplementary Table S3. Whole exome sequencing data of 98 ampullary adenocarcinomas using Illumina multiplexing was obtained^5^. In ampullary adenocarcinoma, there was no alteration of *SFRP1* in DNA sequencing or in copy number. In other cancers, the incidence of *SFRP1* alteration was also low (0.4%–3.0% in these 4 kinds of cancer). The most frequent gene alteration in ampullary cancer was found in *APC* (23%), followed by *CTNNB1* (9%), *RNF43* (8%), *CDH1* (1.9%), and *WNT1* (1.3%). We observe thee co-occurrence of *WNT1-RNF43* alteration in two patients, *APC-RNF43* in two patients, *APC-CDH1* in three patients, and *APC-CTNNB1* in one patient (Supplementary Fig. S3A). The co-occurrence tendency was evaluated by calculating the odds ratio of the number of patients with genetic alterations (Supplementary Table S3). The co-occurrence of *SFRP1* with these five genes was confirmed in pancreatic cancer; however, the correlation was not established in other cancers due to the relatively low alteration rate in the *SFRP1* gene.

### SFRP1 expression in ampullary adenocarcinoma

**Figure 5:**
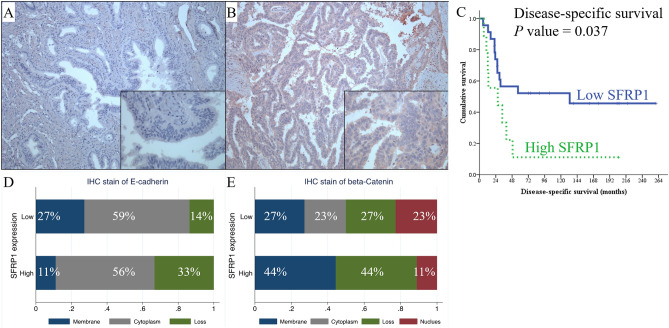
Immunohistochemistry staining of SFRP1 in ampullary adenocarcinoma. (**A**) Negative expression of SFRP1. (**B**) High expression of SFRP1. (**C**) Kaplan–Meier analysis of the impact of SFRP1 expression on disease-specific survival in ampullary adenocarcinoma patients (*P* = 0.037). (**D**) Correlation between localisation of E-cadherin staining and expression of SFRP1 in using immunohistochemistry (IHC). High expression of SFRP1 is slightly correlated with the loss of E-cadherin, and low expression of SFRP1 is correlated with membranous staining of E-cadherin (*P* = 0.362). (**E**) Correlation between the localisation of β-catenin staining and expression of SFRP1 using IHC. High expression of SFRP1 is slightly associated with membranous staining of β-catenin, and low expression of SFRP1 is associated with nuclear staining of β-catenin (*P* = 0.301). IHC, immunohistochemistry; SFRP1, secreted frizzled related protein 1.

We employed immunohistochemistry (IHC) staining to detect the expression of SFRP1 protein in cancer samples to verify the impact of SFRP1 on the survival of ampullary adenocarcinoma patients (Fig. 5A,B). Of the 32 samples analysed, a low level of SFRP1 expression was detected in 23 (72%) and a high level of expression in 9 (28%) patients. The patient cohort was too small for a complete analysis (Table 1). However, the patients with a high expression of SFRP1 tended to have higher risk of early recurrence within 12 months of operation, and increased proportion of peritoneal carcinomatosis (Table 2). The disease-specific survival rate of patients with high SFRP1 expression levels was worse than those with low SFRP1 expression levels (Fig. 5C). The IHC results indicated that the influence of SFRP1 expression in survival of ampullary adenocarcinoma patients was similar to rectal and gastric adenocarcinomas (Figs. 2E,F, and 4E). The result was contrary to the survival analyses determined for pancreatic adenocarcinoma or cholangiocarcinoma (Figs. 3E, 4O,T). Thus, the characteristics of ampullary adenocarcinoma was seemingly prone to certain types of gastrointestinal adenocarcinoma.

**Table 1:** Correlation of histopathological factors and SFRP1 expression detected by IHC staining in patients with ampullary adenocarcinoma.

	SFRP1 expression		*P*
	Low	High	
No. (%)	23 (72%)	9 (28%)	
Age, years, median (range)	66 (36–90)	65 (32–80)	NS
**Sex**			NS
Female	12 (75%)	4 (25%)	
Male	11 (69%)	5 (31%)	
Total bilirubin, mg/dL, median (range)	2.7 (0.5–10.8)	2.3 (0.2–14.4)	NS
Direct bilirubin, mg/dL, median (range)	1.7 (0–9.4)	2.4 (0.1–12.4)	NS
CEA, units/ml, median (range)	2.5 (0.1–21.7)	2.0 (0.9–11.1)	NS
CA125, units/ml, median (range)	13.6 (2.7–44.0)	18.7 (8.3–66.7)	NS
CA199, units/ml, median (range)	44.2 (1.4–1,860)	137.7 (5–2,120)	NS
**Tumor type**			NS
Polypoid	9 (64%)	5 (46%)	
Infiltrative	7 (78%)	2 (22%)	
Mixed	6 (75%)	2 (25%)	
Tumor size, cm, median (range)	3 (0.8–6.5)	2.5 (1.0–8.0)	NS
**Differentiation**			NS
Well	8 (67%)	4 (33%)	
Moderate	12 (80%)	3 (20%)	
Poor	2 (67%)	1 (33%)	
**Tumor stage**			NS
T1	2 (67%)	1 (33%)	
T2	5 (63%)	3 (37%)	
T3	12 (80%)	3 (20%)	
T4	4 (67%)	2 (33%)	
**Lymph node status**			NS
Negative	14 (78%)	4 (22%)	
Positive	9 (75%)	3 (25%)	
**Pancreatic invasion**			NS
Negative	7 (70%)	3 (30%)	
Positive	16 (76%)	5 (24%)	
**AJCC TNM stage**			NS
Stage I	6 (60%)	4 (40%)	
Stage II	12 (86%)	2 (14%)	
Stage III	4 (67%)	2 (33%)	
Stage IV	1 (50%)	1 (50%)	

SFRP1, secreted frizzled related protein 1; NS, not significant; CEA, carcinoembryonic antigen; CA125, cancer antigen 125; CA199, cancer antigen 199; AJCC TNM stage, American Joint Committee on Cancer’s tumor, node, and metastases staging system.

**Table 2:** Correlation between disease recurrence and SFRP1 expression detected by IHC staining in patients with ampullary adenocarcinoma after curative resection.

	Expression of SFRP1		*P*
	Low	High	
No recurrence, n (%)^a^	10 (50%)	1 (13%)	NS
**Recurrence, n (%)**^**a**^	10 (50%)	7 (87%)	
Delayed recurrence (after postoperative 12 months)	4	2	NS
Early recurrence (within postoperative 12 months)	6	5	0.056
**Patterns of recurrence**^**a,b**^			
Liver metastasis	3	3	NS
Local recurrence	5	5	0.061
Peritoneal carcinomatosis	1	3	0.015
Bone metastasis	5	0	NS
Other metastasis^c^	4	0	NS

SFRP1, secreted frizzled related protein 1; NS, not significant.

^a^Excluding two patients who died due to surgical complications and two patients who lost follow-up in NCKUH.

^b^Some patients developed more than one kind of metastases.

^c^Including brain, lung, and ovary metastases.

In our previous study, the loss of β-catenin was correlated with a poor prognosis for patients with ampullary adenocarcinoma^12^. The original dataset correlates with the present study. A high expression of SFRP1 was correlated with the loss of E-cadherin, and a low expression of SFRP1 was correlated with a trend of membranous staining of E-cadherin (*P* = 0.362, Fig. 5D). The high expression of SFRP1 was associated with membranous staining of β-catenin, and low expression of SFRP1 was slightly associated with nuclear staining of β-catenin (*P* = 0.301, Fig. 5E). The patient cohort was too small to determine the statistical significance; however, our data indicated that SFRP1expression was associated with the dysregulation of WNT signalling.

### SFRP1 expression in periampullary adenocarcinoma

**Figure 6:**
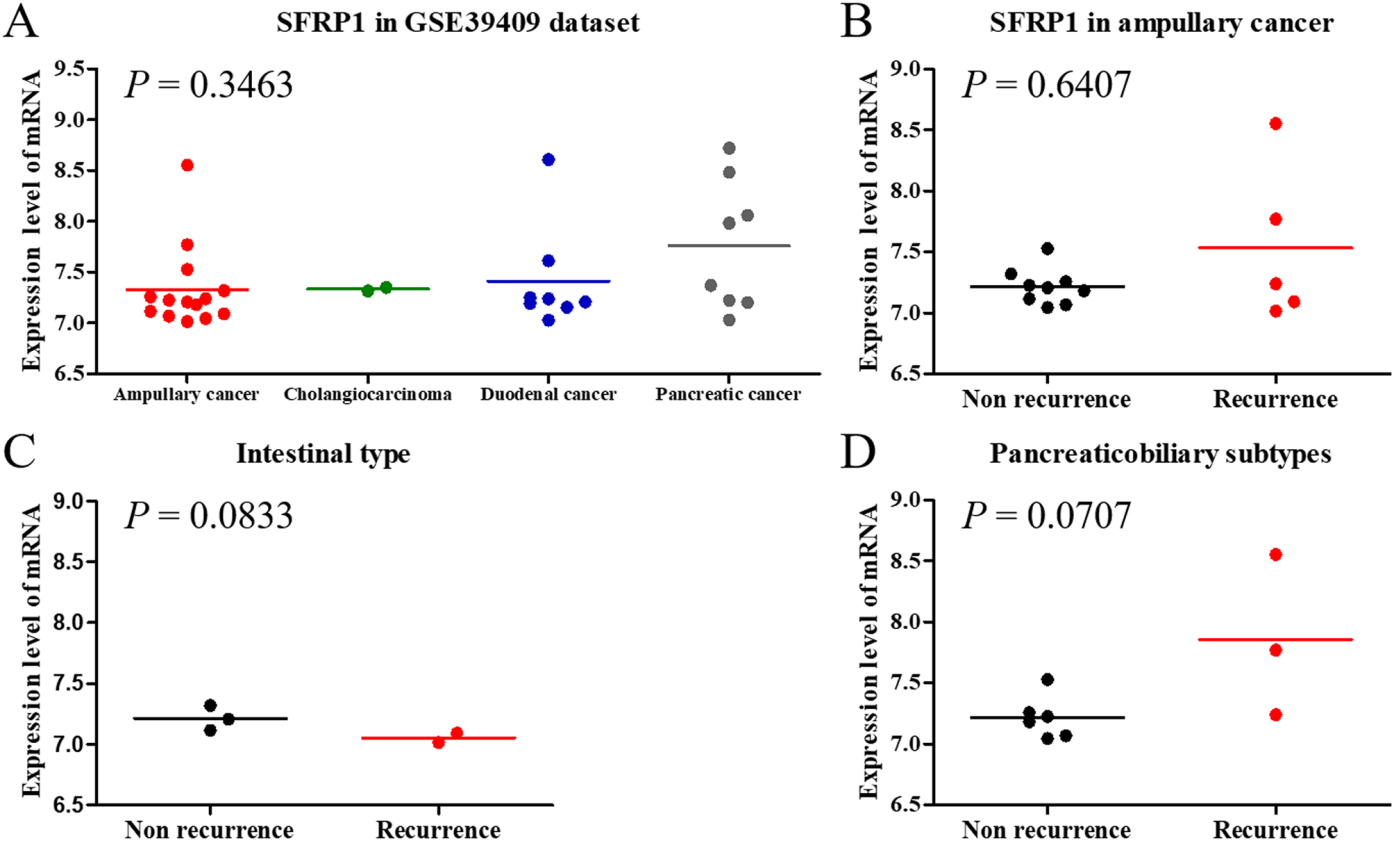
Expression of SFRP1 in microarrays. (**A**) Gene expression level of SFRP1 mRNA in the GEO GSE39409 dataset. Expression of SFRP1 in ampullary adenocarcinoma was lower than other periampullary adenocarcinoma. (**B**) Correlation of SFRP1 mRNA with recurrent status of ampullary adenocarcinoma patients in the GSE39409 dataset. (**C**) Correlation of SFRP1 mRNA with recurrent status of patients with intestinal subtypes. (**D**) Correlation of SFRP1 mRNA with recurrent status of patients with pancreaticobiliary subtypes. The patients with recurrence had a trend of higher ratio of SFRP1 expression. SFRP1, secreted frizzled related protein 1.

Because ampullary adenocarcinoma is a rare cancer, there is minimal bioinformatic information available in public databases. The GSE39409 dataset from the National Center for Biotechnology Information contains data of 14 ampullary adenocarcinoma, two cholangiocarcinoma, eight duodenal adenocarcinoma, and eight pancreatic adenocarcinoma cases^27^. The mRNA expression of SFRP1 was detected using the Affymetrix U133 Plus 2.0 genome array. The log_2_ ratio of SFRP1 expression in cancer vs. normal tissue was calculated. The patients with ampullary adenocarcinoma had median log_2_ ratio of SFRP1 of 7.22 (range, 7.02–8.55). Pancreatic adenocarcinoma patients had a median log_2_ ratio of SFRP1 of 7.68 (range, 7.03–8.72). SFRP1 expression was relatively lower in ampullary adenocarcinoma than in pancreatic adenocarcinoma (Fig. 6A). The expression level of SFRP1 mRNA was similar in patients with a recurrence of ampullary adenocarcinoma (median log_2_ ratio of SFRP1, 7.24; range, 7.02–8.55) and in patients without recurrence (median log_2_ ratio of SFRP1, 7.21; range, 7.02–8.55) (Fig. 6B). SFRP1 expression was not correlated with a recurrent status in patients with intestinal subtype (Fig. 6C). For patients with pancreaticobiliary subtypes, the recurrent patients had a higher level of SFRP1 (Fig. 6D). Expression of WNT1, APC, RNF43, and CDH1 were similar between patients with or without recurrence (Supplementary Figure S4). However, the patients with cancer recurrence showed a higher level of CTNNB1 gene expression than those without recurrence (Supplementary Figure S4D). However, it must be remembered that the number of patients in our study was limited.

### WNT-associated signalling in ampullary adenocarcinoma

**Figure 7:**
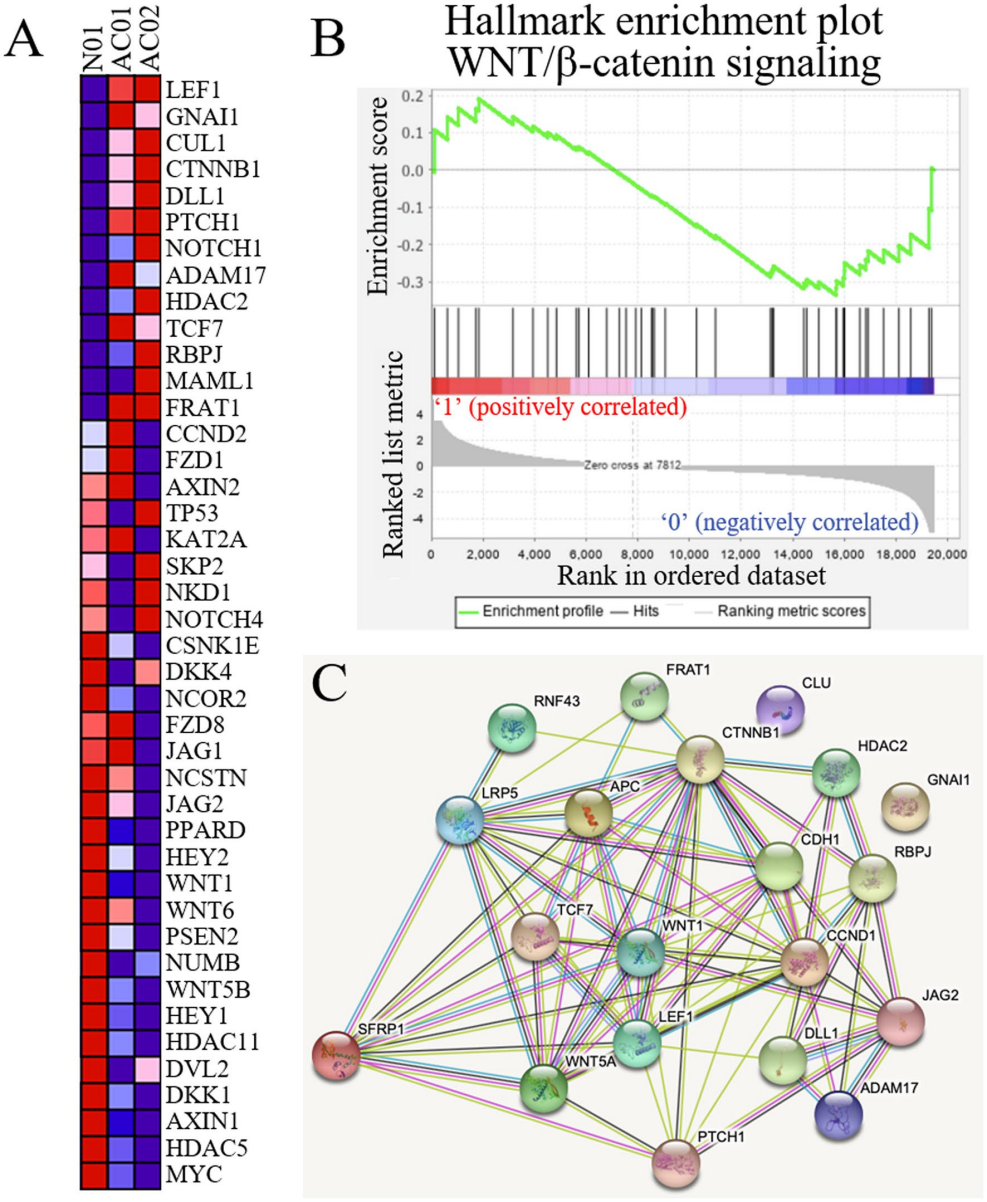
Expression of WNT-associated genes by microarray of primary culture cells. (**A**) Primary culture cells of ampullary adenocarcinoma were analysed by microarray of oligo-chips. A heatmep was generated by comparison with normal duodenal cells (N01) with early and advanced ampullary cancer. Red represents upregulated genes, and blue represents downregulated genes. (**B**) Gene set enrichment analysis (GSEA), using the MSigDB hallmark gene set collection database in primary culture cells, associated with WNT-β-catenin signalling. Enrichment score is shown in the upper third of the graph. Each bar over the middle third represents one gene located in the ranking. Red indicates a positive correlation and blue as a negative correlation. The rank distribution along with the gene list is shown as the grey part in the lower-third of each graph. (**C**) Protein–protein interaction of WNT-associated genes was predicted by STRING software. SFRP1 was linked with multiple genes. N01, primary culture cells from normal duodenum. AC01, primary culture cells from well-differentiated ampullary adenocarcinoma, T1N0, stage IA. AC02, primary culture cells from moderately-differentiated ampullary adenocarcinoma, T2N1, stage IIB. GSEA, gene set enrichment analysis; *SFRP1*, secreted frizzled related protein 1; STRING, search tool for the retrieval of interacting genes.

SFRP1 is one of the extracellular ligands of frizzled receptors. SFRP1 binds to Wnt ligands, which interacts with frizzled receptors in canonical WNT signalling^17^. WNT signalling activation induces nuclear accumulation of β-catenin and switches on the transcription of downstream genes^6^. In our previous study, the loss of β-catenin was correlated with poor prognosis of patients with ampullary adenocarcinoma^12^. Thus, the dysregulation of WNT signalling in ampullary adenocarcinoma was a possibility. Therefore, WNT-associated signalling required consideration to explain the function of SFRP1 in ampullary adenocarcinoma. We collected fresh tissue to establish primary cultured cells and studied the genes that interacted with *SFRP1*. One clone from adjacent duodenal mucosa (N01) and two clones from ampullary adenocarcinomas (AC01 and AC02) were established and analysed with a cDNA microarray. The WNT-related genes were selected to generate heatmap (Fig. 7A). Highly differentiated genes that were upregulated in cancer cells and downregulated in normal cells were identified. *LEF1* (lymphoid enhancer-binding factor 1) was identified as the gene with largest difference and ranked 126th in all 41,000 genes. Gene set enrichment analysis (GSEA) was performed to analyse the WNT signalling (Fig. 7B). The enrichment score was − 0.34 and most WNT-related genes were downregulated in AC01 and AC02 cells. The potential networks of protein–protein interaction of WNT-related genes were drawn using Search Tool for the Retrieval of Interacting Genes (STRING) software (Fig. 7C), which showed that SFRP1 was linked with multiple genes.

**Figure 8:**
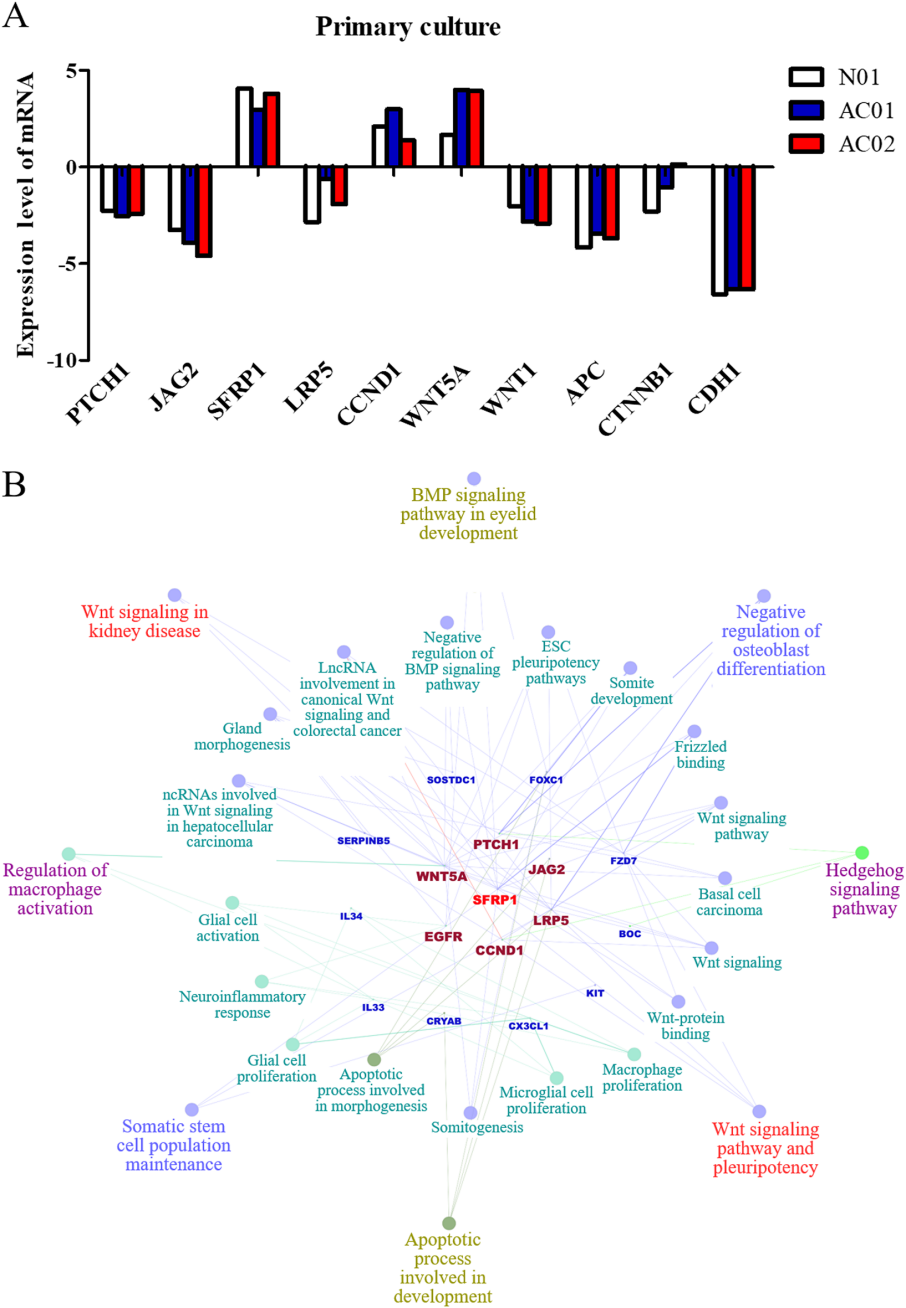
Expression of SFRP1-associated genes by microarray of primary culture cells. (**A**) Primary culture cells of ampullary adenocarcinoma was analysed by oligochip microarray. Most WNT-associated genes were downregulated in these cells. Upregulation of SFRP1 and oncogenes, CCND1 and WNT5A, were detected. (**B**) ClueGo and CluePedia were used to create the network with gene GO terms. *SFRP1* was shown in the centre (red), and the other genes were located in the inner track of concentric circles (dark red). Downstream genes were placed in the middle track (blue), and the phenotypes were placed in the outer two rings. The first inner ring included *PTCH1*, *JAG2*, *LRP5*, *CCND1*, *EGFR* and *WNT5A*. Interaction between *SFRP1* and the first-ring genes was deduced to genes in the middle circle (blue). The correlated phenotypes were presented in the outer two circles. The node size reflected the enrichment of genes in each of the GO terms. The connecting lines showed the correlation between genes and/or pathways. N01, primary culture cells from normal duodenum. AC01, primary culture cells from well-differentiated ampullary adenocarcinoma, T1N0, stage IA. AC02, primary culture cells from moderately-differentiated ampullary adenocarcinoma, T2N1, stage IIB. *APC*, adenomatosis polyposis coli; BMP, bone morphogenetic protein; *BOC*, BOC cell adhesion associated, oncogene regulated; *CCND1*, cyclin D1; *CDH1*, cadherin 1; *CRYAB*, crystallin alpha B; *CTNNB1*, catenin beta 1; *CX3CL1*, C-X3-C motif chemokine ligand 1; *EGFR*, epidermal growth factor receptor; ESC, embryonic stem cell; *FOXC1*, forkhead box C1; *FZD7*, frizzled class receptor 7; *IL33*, interleukin 33; *IL34*, interleukin 34; *JAG2*, jagged canonical notch ligand 2; *KIT*, KIT proto-oncogene, receptor tyrosine kinase; *LRP5*, low-density lipoprotein receptor-related protein 5; *PTCH1*, protein patched 1; *SERPINB5*, serpin family b member 5; *SFRP1*, secreted frizzled related protein 1; *SOSTDC1*, sclerostin domain containing 1; *WNT1*, Wnt family member 1; *WNT5A*, WNT family member 5A.

We chose *SFRP1*-related genes and performed an analysis on these choices using primary culture cells (Fig. 8A). The expression of *SFRP1* was increased in normal and in adenocarcinoma cells. Decreased expression of *PTCH1* (protein: protein patched homolog 1), *JAG2* (protein: jagged canonical notch ligand 2), *LRP5* (protein: low-density lipoprotein receptor-related protein 5), *WNT1* (protein: WNT family member 1), *APC*, *CTNNB1*, and *CDH1* mRNA was detected. Increased expression of *CCND1* (protein: cyclin D1) and *WNT5A* (protein: WNT family member 5A) was showed. The relationship of these genes is shown in Fig. 8B with predicted clinical phenotypes. Crosstalk between WNT, bone morphogenic protein (BMP), and hedgehog signalling was been implicated.

## Discussion

Ampullary adenocarcinoma is a rare malignancy from periampullary region. Because of its characteristics, it is unreasonable to treat ampullary adenocarcinoma patients with identical modalities as other gastrointestinal cancers and personalised therapy should be established. Dysregulation of oncogenic WNT signalling has been reported in ampullary adenocarcinoma. The present study focused on SFRP1, one of the extracellular ligands in the WNT pathway. SFRP1 mRNA expression was detected in 22 types of cancer. The correlation between SFRP1 expression and cancer patients' survival was discordant according to the bioinformatics analyses with Kaplan–Meier Plotter and PrognoScan. Increased methylation of the promoter region of SFRP1 was consistent with low expression of *SFRP1*. There was no alteration of *SFRP1* found on microarray analysis of ampullary adenocarcinoma. We employed IHC staining to study SFRP1 expression in ampullary adenocarcinoma. High SFRP1expression was weakly associated with decreased membranous staining of E-cadherin. High SFRP1 expression was correlated with poor prognosis, early recurrence and peritoneal carcinomatosis of ampullary adenocarcinoma patients. SFRP1 mRNA expression in ampullary adenocarcinoma was lower than in other periampullary adenocarcinomas; however, there was a trend of higher SFRP1 mRNA in ampullary adenocarcinoma patients with recurrence. We performed a cDNA microarray study of primary culture cells from ampullary adenocarcinoma. Increased expression of *SFRP1*, *CCND1* and *WNT 5A* was related to the downregulation of *PTCH1*, *JAG2*, *LRP5, WNT1, APC, CTNNB1,* and *CDH1*. Interaction between these genes may lead to the non-canonical signalling of SFRP1 and result in cancer dissemination.

The ampulla of Vater is the confluent orifice of the pancreatic duct and common bile duct. The growth of ampullary adenocarcinoma derives primarily from the mucosa of the common channel of both ductal systems. The carcinogenesis of ampullary adenocarcinoma is under study, and the activation of WNT signalling is one of the possible mechanisms. The mutation of tumor suppressors (e.g., APC, GSK-3, and AXIN) in the WNT pathway was found to induce nuclear accumulation of β-catenin^9^. The membranous loss of E-cadherin and β-catenin was observed in ampullary adenocarcinoma^8,12^. Aberrant nonmembranous β-catenin expression was observed in intestinal subtype of ampullary cancer^11^. SFRP family proteins are extracellular ligands of frizzled receptors, and the functions of SFRP family proteins differ. Promoter hypermethylation of SFRP1, SFRP2, SFRP4, and SFRP5 is associated with gene suppression and higher cancer risk^28^. However, there are several databases that have shown equivocal correlation between gene expression of SFRP1 and survival of cancer patients. Molecular subtypes of cancer may alter the regulatory system of SFRP1. Intrinsic subtypes of breast cancer are grouped by gene expression patterns and clinical relevance. Smid et al. performed microarray analysis of 344 primary breast tumors. High expression levels of SFRP1 was reported in basal subtype and brain relapse patients with a poor prognosis; however, low expression levels of SFRP1 was revealed in luminal B and bone relapse patients with a relatively better prognosis^29^. SFRP1 overexpression of is detected in human gastric cancer and correlated with lymph node metastasis and poor patient prognosis^26^. Upregulation of SFRP1 is detected in metastatic renal cell carcinoma cell lines and the knockdown of SFRP1 by small interfering RNA inhibits the invasive potential^25^.

In the canonical pathway, SFRP1 interacts with WNT protein and restrains the binding of WNT with frizzled protein, which is the key step of WNT signalling. SFRP1 acts as a WNT inhibitor in canonical pathway. Nevertheless, there are several conflicting reports. The cysteine-rich domain of SFRP1 interacts with frizzled receptors in prostate cancer cells, and the SFRP1/frizzled complexes activate a signal that may lead to the repression of downstream signalling^30^. Treatment with SFRP1 decreases signalling through the Wnt/β-catenin pathway and the increases cell proliferation of prostate epithelial cells^31^. Co-activation of the transforming growth factor-beta (TGF-β) signalling pathway and SFRP1 induces cell proliferation and lung metastasis in mouse model of gastric cancer^26^. SFRP1 activates glycogen synthase kinase 3β (GSK3β) and Rac family small GTPase 1 to interact with TGF-β in promoting cell proliferation, migration, and invasion^32^. Collaboration of TGF-β and BMP, WNT-β-catenin, and hedgehog pathways are critical in the EMT of cancer^33^. Secretion of extracellular SFRP1 establishes crosstalk with adhesion molecules in acute myeloid leukaemia cells and contributes to drug resistance^34^. The hedgehog signalling pathway is upstream of SFRP1, and hedgehog activation increases the transcription of SFRP1^35^. SFRP1 suppresses WNT signalling; concomitantly, hedgehog signalling promotes stemness of cancer cells^36^. SFRP1 is the hinge of WNT- or hedgehog-predominant pathway in cancer, and collaboration of SFRP1 with other signalling pathways is possible. In the present study, we collected data from The Human Protein Atlas, Kaplan–Meier Plotter, PrognoScan and DNMIVD. Survival analysis was equivocally associated with SFRP1 expression. We also used microarray analysis to study SFRP1 and potential interacting molecules. The fresh samples of periampullary adenocarcinoma from the University of Texas MD Anderson Cancer Center (UTMDACC) were analysed by Affymetrix U133 Plus 2.0 Array, and the differentially expressed genes were studied (Fig. 6). The primary culture cells from patients with ampullary neoplasms were established in National Cheng Kung University Hospital (NCKUH) and investigated using the Agilent Human Whole Genome Oligo 4 × 44 K Microarray chip (Figs. 7 and 8). The different origin of mRNA and dissimilar detection platform of the microarrays resulted in direct comparisons between the two datasets being impossible. The expression of SFRP1 mRNA in the UTMDACC dataset was higher in ampullary adenocarcinoma patients with recurrence, especially in those with pancreaticobiliary subtypes. Most WNT-associated genes were downregulated in the GSEA analysis. However, the SFRP1 mRNA in the NCKUH dataset was increased in normal and in cancerous primary culture cells. Additionally, we performed IHC staining to verify expression of SFRP1 protein in ampullary adenocarcinoma. The patients with high SFRP1 staining had a poor prognosis and increased proportion of peritoneal carcinomatosis. The upregulation of *SFRP1* and other interacting genes established a network to connect WNT, BMP, and hedgehog pathways. The present study provided a possible explanation regarding the interaction with different oncogenic pathways in ampullary adenocarcinoma.

Ampullary adenocarcinoma is a heterogeneous cancer with intestinal and pancreaticobiliary subtypes^4^. Molecular signatures are utilised to group patients. Patients with pancreaticobiliary subtypes have a worse prognosis than those with intestinal subtypes^3,4^. The pathologist at NCKUH did not recognise pancreaticobiliary or intestinal subtypes of ampullary adenocarcinoma before 2014, and the present study was executed from September 1994 to August, 2007, before the usage of the classification system at NCKUH. We collected samples from all subtypes of ampullary adenocarcinoma and elucidated the function of SFRP1. The weak point of the present study was the lack of clear classification of subtypes in ampullary adenocarcinoma and minimal amounts of samples. Our results included real world heterogeneity. Moreover, the mRNA microarray from UTMDACC had similar results to our findings, thus the results of the present study could represent the general condition of ampullary adenocarcinoma.

Even though there are plenty of studies regarding the somatic mutation of ampullary adenocarcinoma, the mechanism of carcinogenesis and dissemination is not well understood. The survival of patients with ampullary cancer ranges from 30 to 50%^2,3,37^. Adjuvant chemotherapy for high-risk patients is not associated with improved survival^38^. 5-Fluorouracil-based chemotherapy has some benefit to improve survival, but the results are unsatisfactory^39^. Studying oncogenic pathways will help to identify new therapeutic agents. Dysregulation of the WNT pathway is reported in ampullary cancer^5,7–13^. SFRP family proteins bind to Wnt ligands and frizzled receptors to suppress downstream signalling^15^. The expression of SFRP1 predicts a better prognosis in patients with several kinds of cancer^19–24,28^. However, high expression of SFRP1 is correlated with poor prognosis of patients with ampullary adenocarcinoma in our present study. SFRP1 is a protein with multiple functions. Understanding the network interaction surrounding SFRP1 will help to select candidates for target therapy. Solute SFRP1 proteins should be designed for cancers with SFRP1 as a good predictor, including pancreatic and esophageal adenocarcinoma. Monoclonal anti-SFRP1 antibody is a potential drug for cancers with malignant SFRP1 networks, including gastric and ampullary adenocarcinoma.

## Conclusion

SFRP1 is one of the extracellular ligands of the WNT pathway. SFRP1 expression is detected in various types of human cancer, and its clinical impact differs based on the cancer type. High expression levels of SFRP1 predicts poor prognosis in patients with gastric adenocarcinoma but represents better survival of patients with pancreatic adenocarcinoma. High methylation levels of SFRP1 promoter suppresses gene expression. In patients with ampullary adenocarcinoma, the expression level of SFRP1 is lower than other periampullary adenocarcinomas. However, a high expression level of SFRP1 is correlated with cancer recurrence, peritoneal carcinomatosis and poor prognosis for patients. Understanding SFRP1 expression phenotype in ampullary adenocarcinoma will help in the development of new therapeutic agents.

## Materials and methods

### Bioinformatics analysis

All human proteins are mapped in The Human Protein Atlas (https://proteinatlas.org)^40^. Additionally, cancer information from The Cancer Genome Atlas (TCGA) provides the analysis of 17 primary cancers from ≥ 8,000 patients. RNA expression was calculated and expressed as fragments per kilobase of transcript per million mapped reads. The Kaplan–Meier Plotter is a publically available database of microarray results in the Gene Expression Omnibus (GEO) and clinical outcomes from 21 cancer types (https://kmplot.com/analysis/index.php/)^41^. The expression level of target gene in microarray was calculated. The cutoff point of high or low expression was the median level of gene expression in obtained from RNA sequencing of a particular cancer. The univariate Cox regression analysis and Kaplan–Meier survival analysis were performed to validate the prognostic significance. Overall survival was chosen as the default setting. The survival curve was expressed as an HR and *P*-value. The PrognoScan database collects many publicly available microarray datasets of cancer (https://www.prognoscan.org/)^42^. The patients included in the survival analysis were grouped with the optimal cutoff point in gene expression and chosen with a minimum *P*-value. The datasets were selected if the *P*-value was < 0.05 and the 95% CI of HR was not across 0. DNMIVD provided prognostic models based on DNA methylation and gene expression data from TCGA (https://www.unimd.org/dnmivd/)^43^. The correlation between the methylation of gene promoters and patient survival was also available online.

Datasets of cholangiocarcinoma from GEO were searched, and the selection criteria were set as cholangiocarcinoma, *Homo* species and extrahepatic cancer. The GSE132305 dataset was the newest one, with microarray data from 182 extrahepatic cholangiocarcinomas^44^. The raw data were downloaded and analysed for the expression of *SFRP1* mRNA.

Fresh samples of periampullary adenocarcinoma were obtained from the UTMDACC and analysed using the Affymetrix U133 Plus 2.0 Array. The data were deposited online in GEO (accession number GSE39409) (https://www.ncbi.nlm.nih.gov/geo/query/acc.cgi?acc=GSE39409)^27^. Clinical information and disease status were collected by one of our authors (M.J.O.).

The ClueGo and CluePedia database were operated via Cytoscape software version 3.5.1^45,46^. Selection options of GO pathways, organic layout, and colors of nodes were kept according to default setting. The nodes were drawn according to enrichment of genes in GO pathways. The correlation between genes was calculated using Pearson’s correlation, Spearman’s rank correlation coefficient, distance correlation and maximal information coefficient, according to default settings. The related genes and pathways were linked with lines to build interacting networks.

Multidimensional cancer genomics and datasets were collected in the cBioPortal for Caner Genomics^47,48^. One dataset of ampullary adenocarcinoma, seven datasets of gastric adenocarcinoma, ten datasets of colorectal adenocarcinoma or cancer, five datasets of pancreatic adenocarcinoma, and seven datasets of cholangiocarcinoma were selected. Mutation, deletion, insertion, truncation, fusion and copy number alteration of *SFRP1* and other genes were explored.

GSEA software was applied for enrichment analysis of gene sets that had a common biological function, chromosome location or signal pathways^49^. The primary culture cells from one normal duodenum and two ampullary adenocarcinomas were analysed. The MSigDB hallmark gene set collection was applied for matching molecular signatures^50^. We used STRING v 11.0 software to analyse the protein–protein interactions based on the WNT-related genes. This software contains protein–protein interaction networks from 5,090 organisms, 24.6 million proteins, and over 2,000 million interactions^51^. The *k*-means clustering algorithm was selected to classify proteins into different groups.

### Patient population

Patients diagnosed with resectable ampullary adenocarcinoma from September, 1994, to August, 2007, were enrolled from a total of 98 patients that had received curative resection at NCKUH, Taiwan, during the time period. The demographics and pathological reports were retrospectively reviewed from the medical records. The pathological stage was classified according to the guidelines defined by the American Joint Committee on Cancer Staging Manual, 7th edition^52^. Ampullary adenocarcinoma was diagnosed by gross anatomical location and/or transition of normal–dysplasia–carcinoma from the ampulla of Vater and was made by the pathologist on duty. All the patients underwent regular follow-ups and annual examinations that included physical examinations, laboratory tests, abdominal sonography and/or computed tomography. The follow-up planning was scheduled according to the physician’s specialty and routine practice in Taiwan.

Only patients that had agreed to donate specimens to the Human Biobank in NCKUH were included. Some patients were excluded due to a small tumor size and an inadequate sample amount. All participants gave written informed consent, and the samples were preserved in the Human Biobank. Formalin-fixed paraffin-embedded (FFPE) tumor sections, fresh cancer samples or adjacent normal duodenum samples were obtained and de-identified for use in our study.

Recurrence was defined as metastasis to a different organ (liver, bone, brain, lung or ovary), locoregional recurrence, and peritoneal carcinomatosis. Disease recurrence was clarified as biopsy-proven disease or radiological evidence of recurrence. The disease-specific survival rate was limited to those who died due to ampullary adenocarcinoma, and the time period was recorded from the operation date to date of death.

### IHC staining

The FFPE samples obtained from ampullary adenocarcinoma of 32 patients were in the form of 5-μm thick FFPE sections mounted on slides. The slides were de-paraffinised and rehydrated, and the epitopes were reexposed using a heat retrieval method with an autoclave. The nonspecific activity of peroxidase was blocked, and the slides were treated with monoclonal anti-SFRP1 antibody (GeneTex, Irvine, CA, USA) at 4 °C overnight. The secondary antibody was conjugated with a horseradish peroxidase (HRP)-labelled polymer. We used 3-amino-9-ethylcarbazole (Zymed Laboraotries, San Fancisco, MA, USA) to detect the expression of the HRP substrates. All slides were counterstained with hematoxylin and reviewed by a single researcher (H.P.H.). The intensity of SFRP1 immunoreactivity was defined as negative, weak, moderate or strong. SFRP1 was detected in the cytoplasm of adenocarcinoma cells. A negative control for IHC staining was used a confirmation; this was accomplished by the omitting the primary or secondary antibody. A low level of SFRP1 expression was indicated by a negative or weak expression in IHC staining, and a high level of SFRP1 was indicated by a moderate or strong expression.

### cDNA microarray analysis of primary culture cells

Fresh samples from two ampullary adenocarcinoma patients were collected immediately following curative resection with pancreaticoduodenectomy. A sample of the adjacent mucosa of normal duodenum was collected at least 2 cm apart from the ampulla of Vater in one patient. All the tissues were finely dissected into pieces < 0.2 mm, immersed in Dulbecco’s modified Eagle’s (DMEM) with 10% fetal bovine serum and placed in an incubator at 37 °C with 95% humidity and 5% CO_2_. The media was changed every 2–3 days. After attaining 80% confluence, the cells were trypsinised (0.05% trypsin). Unmovalbe cells were defined as fibroblasts and the floating cells considered as immune cells. After subculture for ≥ 3 passages, the cells from the primary culture were collected for cDNA microarray.

Total RNAs were extracted using TRIzol reagent (Invitrogen, Carlsbad, CA, USA). The RNA integrity of each sample was evaluated on an Agilent 2,100 Bioanalyzer (Agilent, Santa Clara, CA, USA). The absorbance was measured at 260, 230, and 280 nm, and the absorbance (A) ratios (A_260_/A_230_ and A_260_/A_280_) of RNA were determined using a MaestroNano spectrophotometer (Maestrogen, Las Vegas, NV, USA), and the target range was set as 1.8–2.1. During the in vitro transcription process, the RNA was labelled with Cy3 dye (PerkinElmer, Waltham, MA, USA). The Agilent Human Whole Genome Oligo 4 × 44 K Microarray chip (Agilent) was hybridised with Cy-labelled complementary RNA. The scanning wavelength of microarray was set at 535 nm for Cy3. Lowe’s method was used for normalisation of the scanned image. Data analysis was performed using Agilent GeneSpring software (Agilent, Santa Clara, CA, USA).

### Statistical analysis

All statistical analyses were performed using SPSS, v17.0 (SPSS, Chicago, IL, USA). Univariate analysis of categorical variables was performed using Pearson’s chi-squared test. The nonparametric Kruskal–Wallis test was utilised for continuous variables. The disease-specific survival rate was calculated by the Kaplan–Meier method and the log-rank test. The level of statistical significance was set as a *P*-value < 0.5.

## Supplementary information

Supplementary Information 1.

## Data Availability

Correspondence and requests for materials should be addressed to H.P.H.
