# Supplementary material for: Increased expression of secreted frizzled related protein 1 (SFRP1) predicts ampullary adenocarcinoma recurrence
Source: Sci Rep. 2020 Aug 6;10:13255. doi: 10.1038/s41598-020-69899-8 (PMC7413269; doi:10.1038/s41598-020-69899-8)
Supplement: Supplementary file 1 — Supplementary Information 1. [file 41598_2020_69899_MOESM1_ESM.doc]

**Original Research:**

**Increased expression of secreted frizzled related protein 1 (SFRP1) predicts ampullary adenocarcinoma recurrence**

**Authors:**

Li-Chin Cheng^1^, Ying-Jui Chao^2,3^, Michael J. Overman^4^, Chih-Yang Wang^5,6,7,8^, Nam Nhut Phan^9^, Yi-Ling Chen^10^, Tzu-Wen Wang^2^, Hui-Ping Hsu^2,11^*, Yan-Shen Shan^2,3^*, Ming-Derg Lai^5,6,12^

^1^Division of Colorectal Surgery, Department of Surgery, Chi-Mei Medical Center, Tainan, Taiwan

^2^Department of Surgery, National Cheng Kung University Hospital, College of Medicine, National Cheng Kung University, Tainan, Taiwan

^3^Institute of Clinical Medicine, College of Medicine, National Cheng Kung University, Tainan, Taiwan

^4^Department of Gastrointestinal Medical Oncology, The University of Texas M. D. Anderson Cancer Center, Houston, Texas, United States of America

^5^Department of Biochemistry and Molecular Biology, College of Medicine, National Cheng Kung University, Tainan, Taiwan

^6^Institute of Basic Medical Sciences, College of Medicine, National Cheng Kung University, Tainan, Taiwan.

^7^Ph.D. Program for Cancer Molecular Biology and Drug Discovery, College of Medical Science and Technology, Taipei Medical University, Taipei, Taiwan.

^8^Graduate Institute of Cancer Biology and Drug Discovery, College of Medical Science and Technology, Taipei Medical University, Taipei, Taiwan

^9^NTT Institute of Hi-Technology, Nguyen Tat Thanh University, Ho Chi Minh City, Vietnam

^10^Senior Citizen Service Management, Chia-Nan University of Pharmacy and Science, Tainan, Taiwan

^11^Department of Biostatistics, Vanderbilt University Medical Center, Nashville, Tennessee, United States of America

^12^Center for Infectious Diseases and Signaling Research, College of Medicine, National Cheng Kung University, Tainan, Taiwan

*Corresponding author:

Hui-Ping Hsu

Address: No. 138, Sheng-Li Rd. Tainan City, 70403, Taiwan

E-mail: [hphsu@mail.ncku.edu.tw](mailto:hphsu@mail.ncku.edu.tw)

Tel.: +886-6-2353535#5272

Fax: +886-6-2766676

Yan-Shen Shan

Address: No. 138, Sheng-Li Rd. Tainan City, 70403, Taiwan

E-mail: [ysshan@mail.ncku.edu.tw](mailto:ysshan@mail.ncku.edu.tw)

Tel.: +886-6-2353535#5182

Fax: +886-6-2766676

| **Supplementary Table S1.** **PrognoScan* microarray analysis of the prognostic value of SFRP1 in human cancer.** High SFRP1 gene expression predicted poor prognosis in these datasets. | | | | | | | | | | | | | | |
| --- | --- | --- | --- | --- | --- | --- | --- | --- | --- | --- | --- | --- | --- | --- |
| Dataset | Cancer type | End point | Cohort | Contributor | Array type | Probe id | N | Cut  point | Minimum *P*-value | Corrected *P*-value | ln (HR-high/HR-low) | Cox *P*-value | ln (HR) | HR  [95% C.I.] |
| GSE13507 | Bladder cancer | OS | CNUH | Kim | Human-6 v2 | ILMN_1775728 | 165 | 0.75 | 0.00 | 0.03 | 0.81 | 0.02 | 0.19 | 1.21  [1.03 - 1.41] |
| GSE13507 | Bladder cancer, transitional cell carcinoma | DSS | CNUH | Kim | Human-6 v2 | ILMN_1775728 | 165 | 0.61 | 0.00 | 0.01 | 1.30 | 0.01 | 0.25 | 1.29  [1.05 - 1.57] |
| GSE4412-GPL97 | Brain cancer, glioma | OS | UCLA (1996-2003) | Freije | HG-U133B | 228413_s_at | 74 | 0.74 | 0.00 | 0.02 | 0.97 | 0.01 | 0.80 | 2.23  [1.26 - 3.96] |
| GSE2837 | Head and neck cancer, squamous cell carcinoma | RFS | VUMC, VAMC, UTMDACC (1992-2005) | Chung | U133_X3P | g8400731_3p_a_at | 28 | 0.89 | 0.00 | 0.01 | 2.73 | 0.01 | 0.74 | 2.10  [1.25 - 3.54] |
| jacob-00182-CANDF | Lung adenocarcinoma | OS | CAN/DF | Shedden | HG-U133A | 202036_s_at | 82 | 0.87 | 0.00 | 0.04 | 1.25 | 0.02 | 0.75 | 2.12  [1.13 - 3.97] |
| GSE9891 | Ovarian cancer | OS | AOCS, RBH, WH, NKI-AVL (1992-2006) | Tothill | HG-U133_Plus_2 | 202036_s_at | 278 | 0.89 | 0.00 | 0.02 | 0.84 | 0.02 | 0.15 | 1.16  [1.02 - 1.31] |
| *www.abren.net/PrognoScan/index.html.  Abbreviations: SFRP1, secreted frizzled related protein 1; CI, confidence interval; DFS, disease-free survival; DMFS, distant metastasis free survival; DRFS, distant recurrence free survival; DSS, disease specific survival; HR, hazard ratio; RFS, recurrence free survival; OS, overall survival. | | | | | | | | | | | | | | |

| **Supplementary Table S2.** **PrognoScan* microarray analysis of the prognostic value of SFRP1 in human cancer.** Low SFRP1 gene expression predicted poor prognosis in these datasets. | | | | | | | | | | | | | | |
| --- | --- | --- | --- | --- | --- | --- | --- | --- | --- | --- | --- | --- | --- | --- |
| Dataset | Cancer type | End point | Cohort | Contributor | Array type | Probe id | N | Cut-point | Minimum p-value | Corrected p-value | ln (HR-high/HR-low) | Cox p-value | ln (HR) | HR  [95% C.I.] |
| GSE12093 | Breast cancer | DMFS | IO, NCI, TUM, CCF (1992-2000) | Zhang | HG-U133A | 202037_s_at | 136 | 0.31 | 0.00 | 0.00 | -1.73 | 0.00 | -0.76 | 0.47  [0.29 - 0.76] |
| GSE2990 | Breast cancer | DMFS | Uppsala, Oxford | Sotiriou | HG-U133A | 202037_s_at | 54 | 0.43 | 0.00 | 0.02 | -2.27 | 0.01 | -0.51 | 0.60  [0.41 - 0.89] |
| GSE2990 | Breast cancer | RFS | Uppsala, Oxford | Sotiriou | HG-U133A | 202037_s_at | 62 | 0.34 | 0.00 | 0.03 | -1.54 | 0.00 | -0.45 | 0.64  [0.47 - 0.87] |
| GSE3494-GPL96 | Breast cancer | DSS | Uppsala (1987-1989) | Miller | HG-U133A | 202037_s_at | 236 | 0.37 | 0.00 | 0.01 | -0.93 | 0.02 | -0.35 | 0.71  [0.53 - 0.94] |
| GSE1456-GPL96 | Breast cancer | DSS | Stockholm (1994-1996) | Pawitan | HG-U133A | 202036_s_at | 159 | 0.13 | 0.00 | 0.02 | -1.28 | 0.02 | -0.31 | 0.73  [0.57 - 0.95] |
| GSE1456-GPL96 | Breast cancer | RFS | Stockholm (1994-1996) | Pawitan | HG-U133A | 202036_s_at | 159 | 0.29 | 0.00 | 0.01 | -1.06 | 0.00 | -0.31 | 0.73  [0.59 - 0.91] |
| GSE4922-GPL96 | Breast cancer | DFS | Uppsala (1987-1989) | Ivshina | HG-U133A | 202037_s_at | 249 | 0.35 | 0.00 | 0.00 | -0.81 | 0.01 | -0.28 | 0.76  [0.61 - 0.94] |
| GSE3494-GPL96 | Breast cancer | DSS | Uppsala (1987-1989) | Miller | HG-U133A | 202036_s_at | 236 | 0.13 | 0.00 | 0.01 | -1.08 | 0.03 | -0.25 | 0.78  [0.62 - 0.98] |
| GSE4922-GPL96 | Breast cancer | DFS | Uppsala (1987-1989) | Ivshina | HG-U133A | 202036_s_at | 249 | 0.12 | 0.00 | 0.01 | -0.91 | 0.02 | -0.21 | 0.81  [0.68 - 0.96] |
| GSE31210 | Lung adenocarcinoma | OS | NCCRI | Okayama | HG-U133_Plus_2 | 228413_s_at | 204 | 0.12 | 0.00 | 0.02 | -1.29 | 0.02 | -0.47 | 0.63  [0.42 - 0.92] |
| GSE31210 | Lung adenocarcinoma | RFS | NCCRI | Okayama | HG-U133_Plus_2 | 228413_s_at | 204 | 0.12 | 0.00 | 0.00 | -1.26 | 0.00 | -0.42 | 0.65  [0.49 - 0.88] |
| GSE31210 | Lung adenocarcinoma | RFS | NCCRI | Okayama | HG-U133_Plus_2 | 202036_s_at | 204 | 0.39 | 0.00 | 0.03 | -0.88 | 0.01 | -0.34 | 0.71  [0.54 - 0.93] |
| GSE30929 | Liposarcoma | DRFS | MSKCC (1993-2008) | Gobble | HG-U133A | 202035_s_at | 140 | 0.14 | 0.00 | 0.03 | -1.05 | 0.02 | -0.23 | 0.79  [0.65 - 0.97] |
| *www.abren.net/PrognoScan/index.html.  Abbreviations: SFRP1, secreted frizzled related protein 1; CI, confidence interval; DFS, disease-free survival; DMFS, distant metastasis free survival; DRFS, distant recurrence free survival; DSS, disease specific survival; HR, hazard ratio; RFS, recurrence free survival; OS, overall survival. | | | | | | | | | | | | | | |

| **Supplementary Table S3.** Alteration of *SFRP1* and WNT-associated genes using the cBioPortal platform. One dataset of ampullary cancer, seven datasets of gastric cancer, ten datasets of colorectal cancer, five datasets of pancreatic cancer, and seven datasets of cholangiocarcinoma were included. There was no alteration, mutation, nor copy number change of *SFRP1* gene in ampullary cancer. Co-occurrence of genetic alterations in *SFRP1* with *WNT1*, *APC*, *RNF43*, *CTNNb1* and *CDH1* was detected in pancreatic cancer. | | | | | | | | | | |
| --- | --- | --- | --- | --- | --- | --- | --- | --- | --- | --- |
| Gene A | Gene B | Diagnosis | Alteration in neither A nor B genes | Alterations in gene A but not in gene B | Alterations in gene B but not in gene A | Alterations in both A and B genes | Log_2_ Odds Ratio | *P* value | *Q* value | Tendency |
| SFRP1 | WNT1 | Ampullary cancer | 158 | 0 | 2 | 0 | > 3 | 1 | 1 | Co-occurrence |
|  |  | Gastric cancer | 1302 | 46 | 32 | 0 | < -3 | 0.334 | 0.417 | Mutual exclusivity |
|  |  | Colorectal cancer | 1704 | 58 | 23 | 4 | 2.353 | 0.013 | 0.021 | Co-occurrence |
|  |  | Pancreatic cancer | 897 | 15 | 4 | 2 | > 3 | 0.005 | 0.006 | Co-occurrence |
|  |  | Cholangiocarcinoma | 235 | 1 | 1 | 0 | < -3 | 0.996 | 1 | Mutual exclusivity |
| SFRP1 | APC | Ampullary cancer | 124 | 0 | 36 | 0 | > 3 | 1 | 1 | Co-occurrence |
|  |  | Gastric cancer | 1149 | 42 | 185 | 4 | -0.758 | 0.222 | 0.333 | Mutual exclusivity |
|  |  | Colorectal cancer | 566 | 16 | 1161 | 46 | 0.487 | 0.155 | 0.194 | Co-occurrence |
|  |  | Pancreatic cancer | 887 | 15 | 14 | 2 | > 3 | 0.033 | 0.042 | Co-occurrence |
|  |  | Cholangiocarcinoma | 231 | 0 | 5 | 1 | > 3 | 0.025 | 0.38 | Co-occurrence |
| SFRP1 | RNF43 | Ampullary cancer | 147 | 0 | 13 | 0 | > 3 | 1 | 1 | Co-occurrence |
|  |  | Gastric cancer | 1202 | 39 | 132 | 7 | 0.709 | 0.173 | 0.288 | Co-occurrence |
|  |  | Colorectal cancer | 1560 | 52 | 167 | 10 | 0.845 | 0.079 | 0.107 | Co-occurrence |
|  |  | Pancreatic cancer | 848 | 15 | 53 | 2 | 1.093 | 0.271 | 0.271 | Co-occurrence |
|  |  | Cholangiocarcinoma | 232 | 1 | 4 | 0 | < -3 | 0.983 | 1 | Mutual exclusivity |
| SFRP1 | CTNNB1 | Ampullary cancer | 146 | 0 | 14 | 0 | > 3 | 1 | 1 | Co-occurrence |
|  |  | Gastric cancer | 1230 | 42 | 104 | 4 | 0.172 | 0.492 | 0.492 | Co-occurrence |
|  |  | Colorectal cancer | 1627 | 53 | 100 | 9 | 1.466 | 0.011 | 0.021 | Co-occurrence |
|  |  | Pancreatic cancer | 888 | 13 | 13 | 4 | > 3 | <0.001 | <0.001 | Co-occurrence |
|  |  | Cholangiocarcinoma | 236 | 1 | 0 | 0 | > 3 | 1 | 1 | Co-occurrence |
| SFRP1 | CDH1 | Ampullary cancer | 157 | 0 | 3 | 0 | > 3 | 1 | 1 | Co-occurrence |
|  |  | Gastric cancer | 1193 | 40 | 141 | 6 | 0.344 | 0.365 | 0.421 | Co-occurrence |
|  |  | Colorectal cancer | 1672 | 59 | 55 | 3 | 0.628 | 0.326 | 0.349 | Co-occurrence |
|  |  | Pancreatic cancer | 898 | 14 | 3 | 3 | > 3 | <0.001 | <0.001 | Co-occurrence |
|  |  | Cholangiocarcinoma | 231 | 1 | 5 | 0 | < -3 | 0.979 | 1 | Mutual exclusivity |
| Odds ratio = (Number of samples with alteration in neither A nor B genes * Number of samples with alterations in both genes) / (Number of samples with alterations in gene B but not in gene A * Number of samples with alterations in gene B but not in gene A).  *P* value was derived from one-sided Fisher Exact Test. *Q* value was derived from Benjamini-Hochberg FDR correction procedure.  Tendency towards co-occurrence while Log_2_ Odds Ratio > 0. Tendency towards mutual exclusivity while Log_2_ Odds Ratio ≤ 0. | | | | | | | | | | |


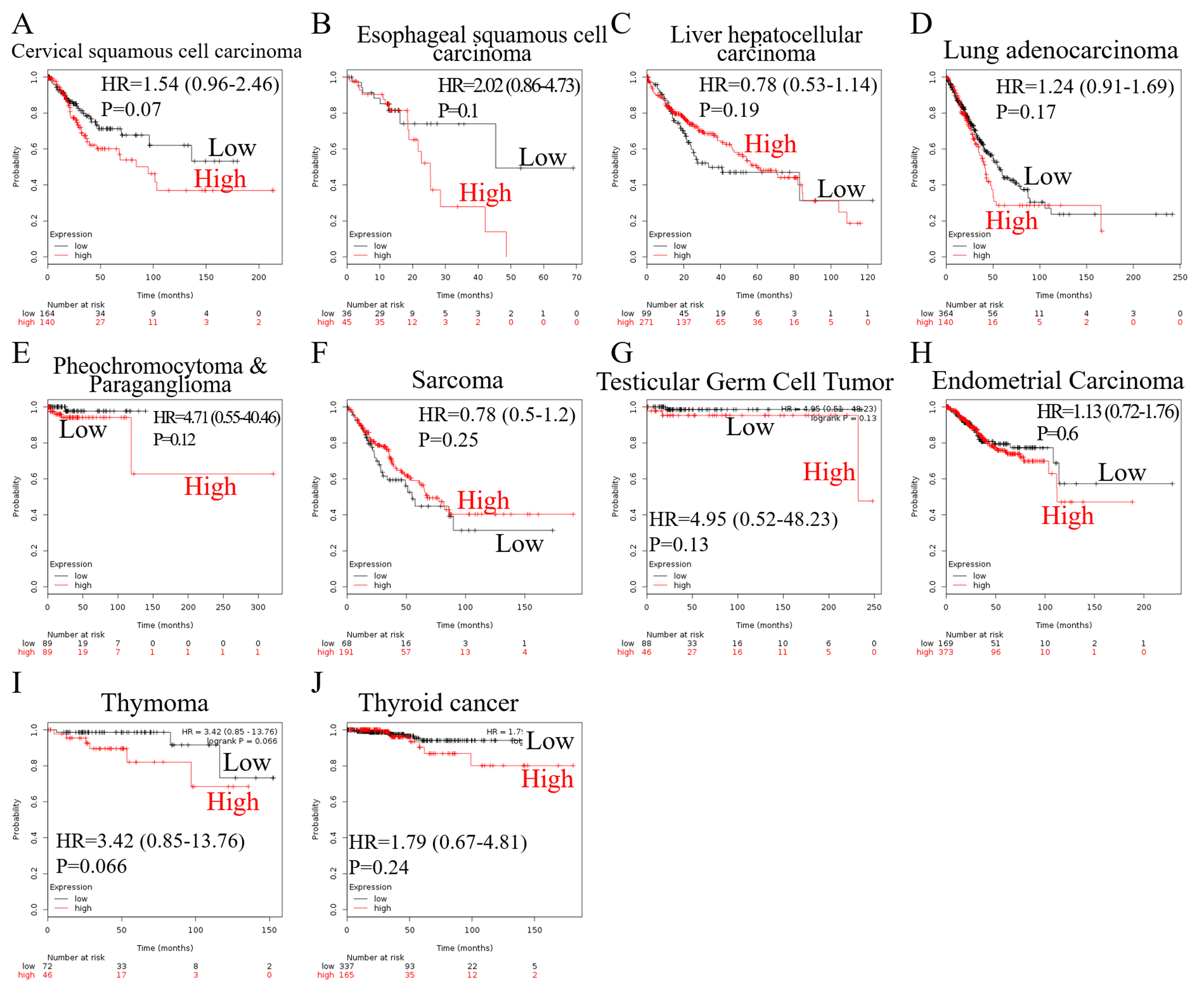


**Supplementary Figure S1.** **Assessment of the effect of SFRP1 mRNA by Kaplan–Meier Plotter.** SFRP1 expression was not correlated with prognosis in patients with (A) cervical squamous cell carcinoma, (B) oesophageal squamous cell carcinoma, (C) liver hepatocellular carcinoma, (D) lung adenocarcinoma, (E) pheochromocytoma and paraganglioma, (F) sarcoma, (G) testicular germ cell tumour, (H) endometrial carcinoma, (I) thymoma, and (J) thyroid cancer. Abbreviations: SFRP1, secreted frizzled related protein 1; HR, hazard ratio.


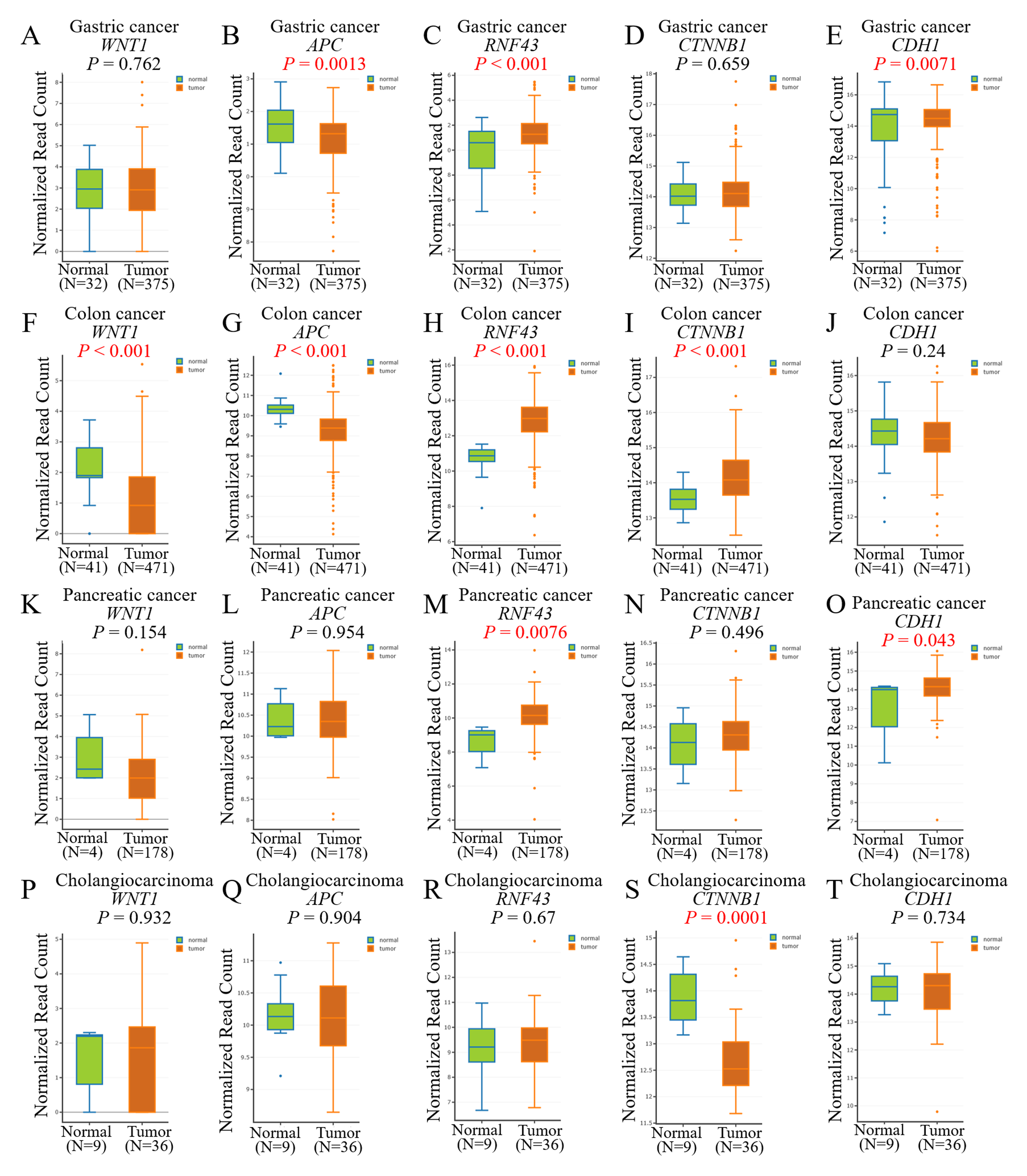


**Supplementary Figure S2. Expression of WNT-related genes in the DNA methylation interactive visualisation database.** Five different profiles of information were shown in four types of cancer. (A), (F), (K), (P) Gene expression of *WNT1* in RNA-sequencing read count. (B), (G), (L), (Q) Expression of *APC* gene. (C), (H), (M), (R) Expression of *RNF43* gene. (D), (I), (N), (S) Expression of *CTNNB1* gene. (E), (J), (O), (T) Expression of *CDH1* gene. (A), (B), (C), (D), (E) Gastric cancer. (F), (G), (H), (I), (J) Colon cancer. (K), (L), (M), (N), (O) Pancreatic cancer. (P), (Q), (R), (S), (T) Cholangiocarcinoma. Abbreviations: APC, adenomatosis polyposis coli; CDH1, cadherin 1; CTNNB1, catenin beta 1; RNF43, ring finger protein 43; WNT1, WNT family member 1.


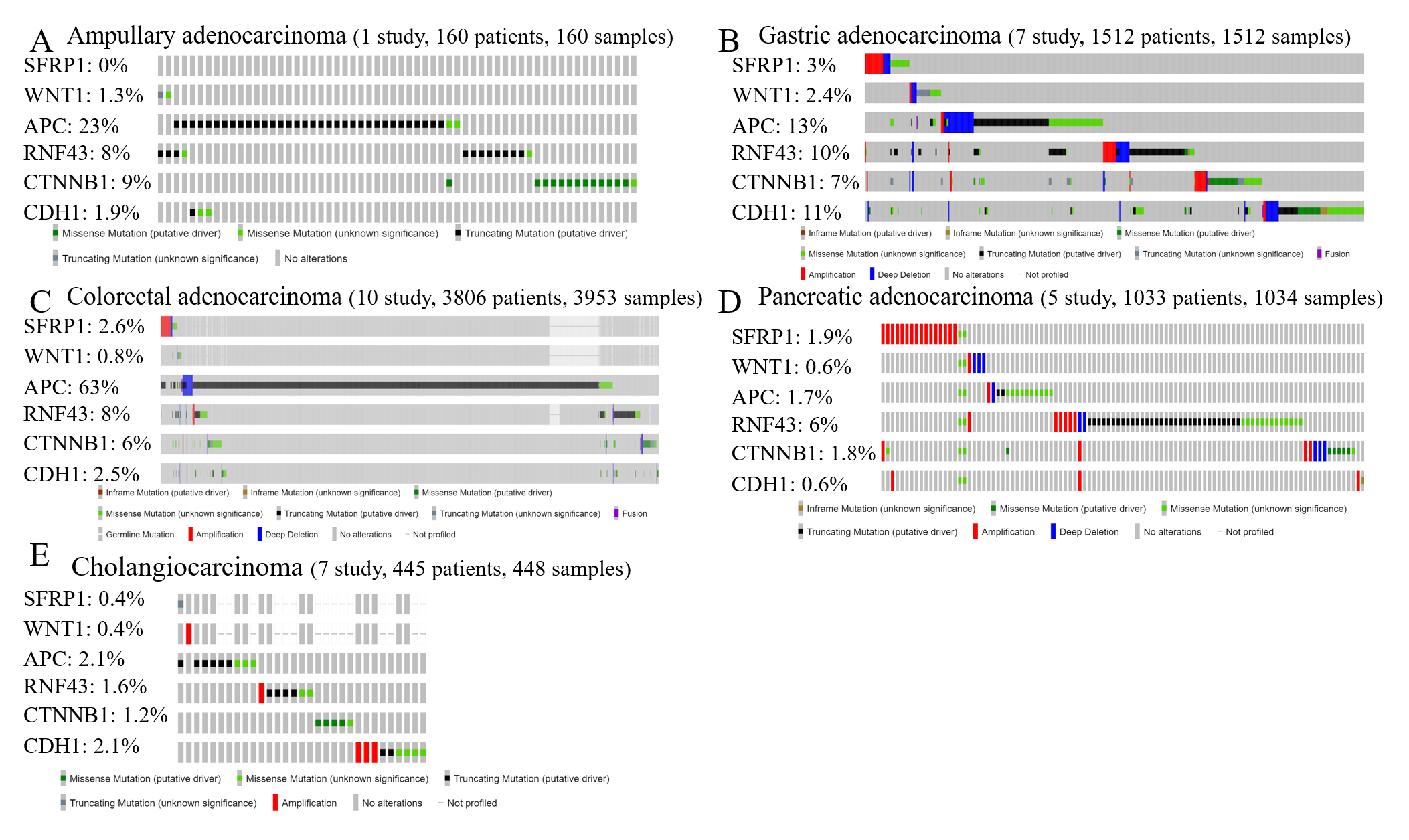


**Supplementary Figure S3. Mutation of WNT-related genes in the cBioPortal platform.** Different kinds of mutations were collated. The percentage after gene name represents the total number of all types. (A) Ampullary adenocarcinoma, (B) gastric adenocarcinoma, (C) colon adenocarcinoma, (D) pancreatic adenocarcinoma, and (E) cholangiocarcinoma.


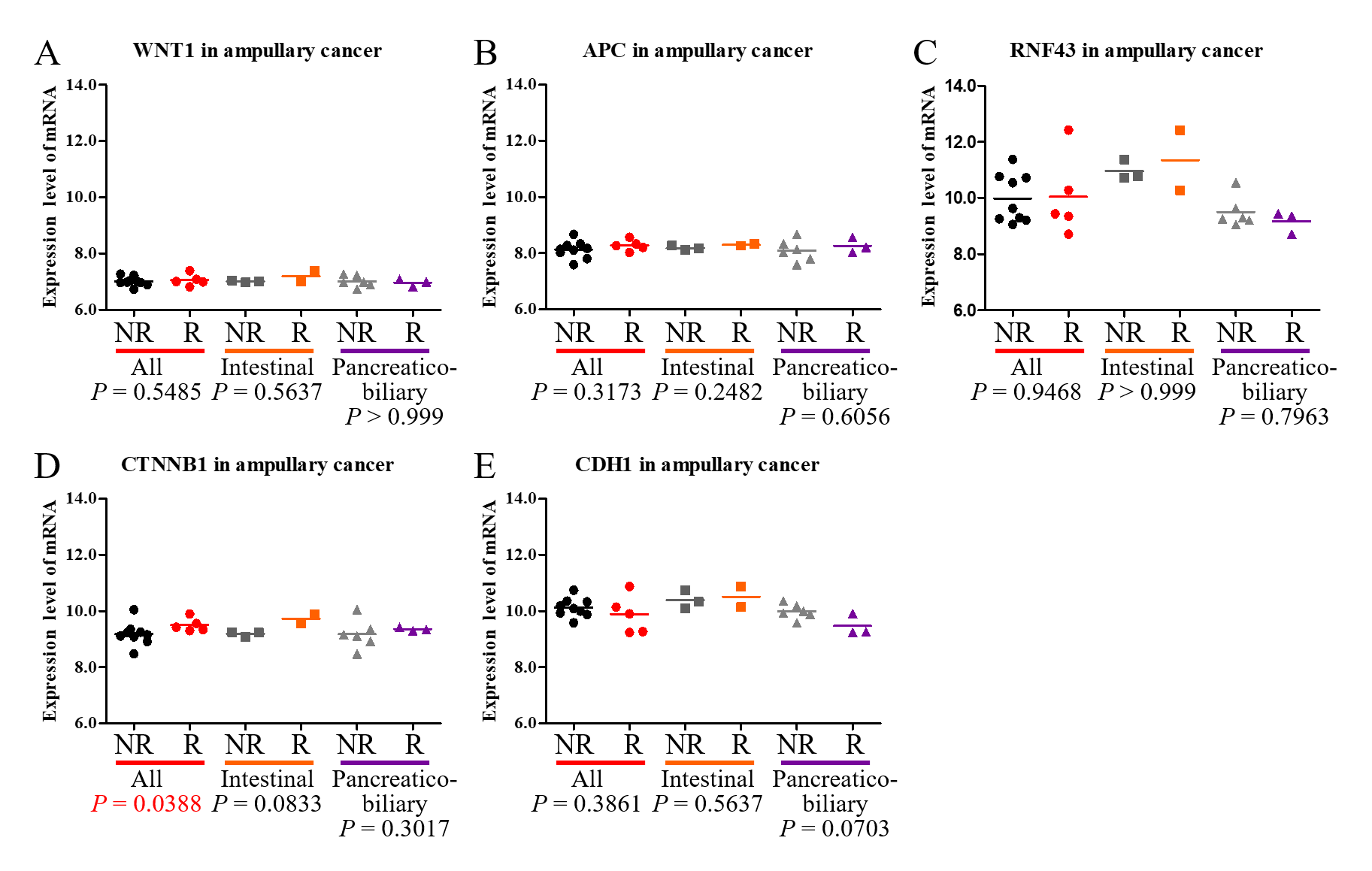


**Supplementary Figure S4. Expression of WNT-related genes in microarrays.** Gene expression of different WNT-related genes was examined in the GEO GSE39409 dataset. (A) *WNT*, (B) *APC*, (C) *RNF43*, (D) *CTNNB1* (protein: β-catenin), and (E) *CDH1* (protein: E-cadherin). Abbreviations: APC, adenomatosis polyposis coli; CDH1, cadherin 1; CTNNB1, catenin beta 1; NR, no recurrence; R, recurrence; RNF43, ring finger protein 43; WNT1, WNT family member 1.
